# Nanospiked Cellulose Gauze That Attracts Bacteria with Biomolecules for Reducing Bacterial Load in Burn Wounds

**DOI:** 10.1021/acs.nanolett.4c05773

**Published:** 2025-01-13

**Authors:** Yuuki Hata, Hiromi Miyazaki, Sayaka Okamoto, Takeshi Serizawa, Shingo Nakamura

**Affiliations:** † Department of Chemical Science and Engineering, School of Materials and Chemical Technology, Institute of Science Tokyo, 2-12-1-H-121 Ookayama, Meguro-ku, Tokyo 152-8550, Japan; ‡ Division of Biomedical Engineering, National Defense Medical College Research Institute, 3-2 Namiki, Tokorozawa-shi, Saitama 359-8513, Japan

**Keywords:** Cello-oligosaccharide, self-assembly, nanostructured surface, wound dressing, bacterial adhesion

## Abstract

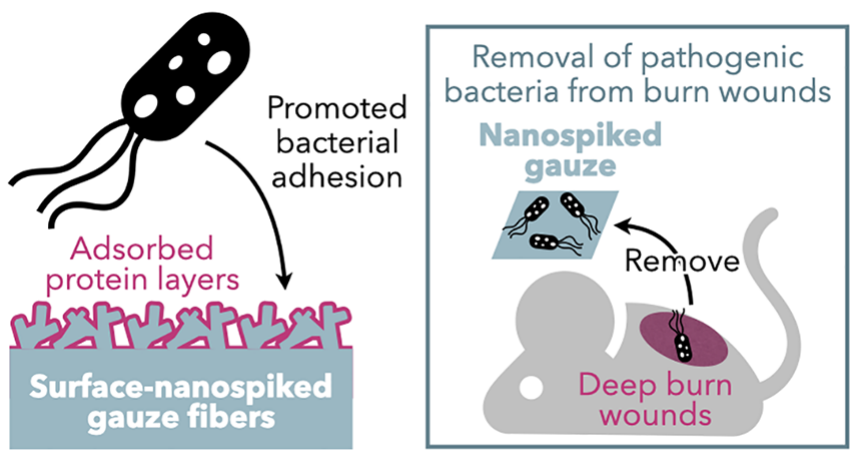

Nanostructuring surfaces is an emergent strategy to endow materials with abilities to combat pathogenic bacteria. Nevertheless, it remains challenging to create nanospike structures on the curved surfaces of polymer materials, including gauze and other microfibrous medical materials. Additionally, the effects of nanostructured surfaces on bacteria in the presence of proteins and in vivo remain largely unexplored. Herein, we demonstrated the decoration of gauze microfiber surfaces with nanospike structures via the self-assembly of cello-oligosaccharides and investigated the effects of the nanospiked gauze on bacteria in the presence of proteins. The nanospiked gauze had low bacterial adhesion properties in the absence of proteins, whereas in the presence of proteins, it promoted bacterial adhesion. Analyses suggested that the adsorbed protein layers on the nanospikes were involved in the promoted bacterial adhesion. Furthermore, the bacterial adhesion-promoting effects were exploited to remove pathogenic bacteria from burn wounds with exudate containing proteins using the nanospiked gauze.

Infectious diseases have consistently been emphasized as a priority issue in global public health and are a significant contributor to the global health burden. A recent study estimated that approximately 13.7 million global deaths in 2019 were attributed to infections, meaning that infections were involved in more than 20% of the all global deaths for that year.^1^ Moreover, the problem of antimicrobial resistance is growing; drug-resistant pathogens are becoming more prevalent globally, while the development of novel antibiotics is declining. A recent study estimated that in 2021, 4.71 million deaths were associated with bacterial antimicrobial resistance, which included 1.14 million deaths attributable to bacterial antimicrobial resistance.^2^ Moreover, it was forecasted that the antimicrobial resistance burden will increase to 8.22 million associated deaths and 1.91 million attributable deaths in 2050. Therefore, it is imperative to develop novel approaches to address the issues of infectious diseases.

Nanostructuring surfaces is an emergent strategy to endow materials with abilities to combat bacteria and other pathogens.^3−6^ While surface chemistry is largely responsible for the properties and functionalities of medical materials,^7−9^ studies have revealed that nanospikes, nanopillars, and other nanostructures influence the adhesion and viability of bacterial cells on material surfaces depending on their shapes, dimensions, hydrophobicity/hydrophilicity, and other structural properties. Some types of nanostructures have low bacterial adhesion properties due to the small contact area between the nanostructures and bacterial cells.^10,11^ Other nanostructures can kill bacteria by mechanically disrupting the cells.^5,12^ Consequently, antibiofouling or bactericidal materials have been created through the nanostructuring of material surfaces. Nanostructuring methods reported to date include reactive-ion etching^13,14^ or chemical etching^15−17^ of silicon, thermal^18^ or hydrothermal^19^ oxidation of titanium alloys, plasma etching of polymers,^20^ self-assembly of copolymers,^21^ and replica molding of poly(dimethylsiloxane)^22^ or hydrophilic polymer hydrogels.^23^ In this context, it remains challenging to create nanospike structures on polymer surfaces with curvature despite the widespread use of gauze, surgical masks, sutures, and other microfibrous polymer materials in medicine. Another largely unexplored aspect of nanostructured antibacterial materials is their effects on bacteria in the presence of proteins and other biomolecular species and in vivo.^19,24^

In this study, we demonstrated the nanostructuring of medical gauze via the self-assembly of cello-oligosaccharides and investigated the effects of the resultant nanospiked gauze on bacteria in protein solutions in vitro and in burn wounds in vivo (Figure 1). Our recent report demonstrated a one-pot nanostructuring process for constructing nanospike structures on paper via the self-assembly of cello-oligosaccharides produced by partial hydrolysis of the raw paper materials.^25^ Filter paper with a high cellulose content was desirable as the raw paper material, whereas common paper (e.g., paper towel) generated unknown impurities due to side reactions during the partial hydrolysis reaction for cello-oligosaccharide production. Therefore, in this study, preprepared cello-oligosaccharides were used for the nanostructuring of medical gauze (Figure 1a). Specifically, high-purity cellulose Avicel PH-101 was hydrolyzed in aqueous phosphoric acid solutions to prepare cello-oligosaccharides. The preprepared cello-oligosaccharides were allowed to self-assemble in medical gauze, yielding surface-nanospiked gauze. We hypothesized that nanospike structures composed of cello-oligosaccharides should influence bacterial adhesion to gauze microfibers. Thus, bacterial adhesion behavior on the nanospiked gauze was investigated in vitro in the absence and presence of crowding proteins (Figure 1b). Moreover, a deep burn wound model in mice was used to explore the potential of nanospiked gauze for infection control (Figure 1c).

**Figure 1:**
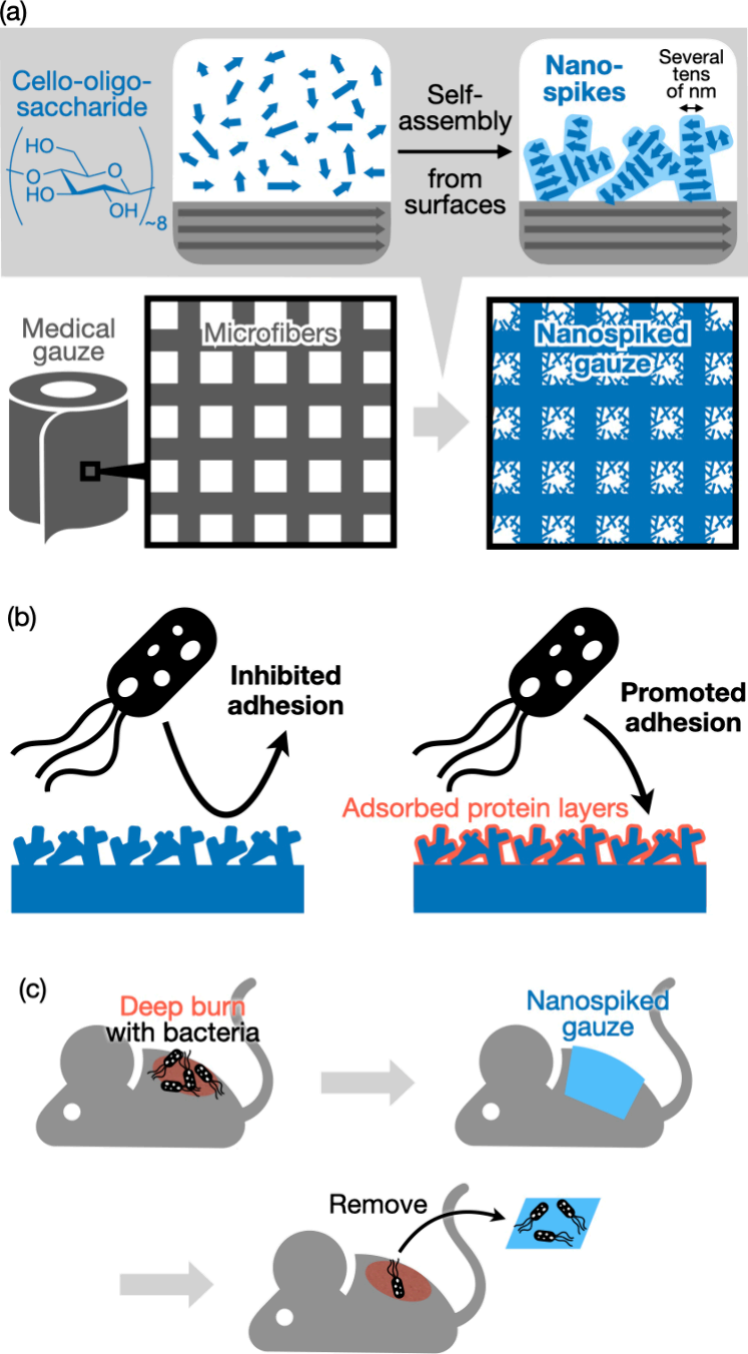
Schematic illustration of this study. (a) The nanostructuring process for medical gauze via the self-assembly of cello-oligosaccharides. (b) Bacterial adhesion behavior on surfaces of the nanospiked gauze. While the nanospikes inhibited bacterial adhesion in clean buffer solutions, in the presence of proteins and other biomolecules, the nanospikes rather promoted bacterial adhesion probably due to the formation of adsorbed protein layers on their surfaces. (c) The removal of pathogenic bacteria from deep burn wounds, which produce exudate containing proteins, using the nanospiked gauze by exploiting the bacterial adhesion-promoting effects.

Cello-oligosaccharides were prepared by the hydrolysis of cellulose in 85% phosphoric acid solutions.^26−28^ The reaction was performed at 45 °C for 20 h and then quenched by adding water. The mass spectrum of the products is shown in Figure S1. Degree of polymerization (DP) of the prepared cello-oligosaccharides ranged from approximately 6 to 17 with a maximum intensity at DP 8. No peaks other than unmodified cello-oligosaccharides were observed in the mass spectra, indicating minimal side reactions during the acid hydrolysis reaction for high-purity cellulose and successful preparation of cello-oligosaccharides.

Cello-oligosaccharides were allowed to self-assemble at a concentration of 1% (w/v) (unless otherwise stated) in a medical gauze. Specifically, the preprepared cello-oligosaccharides were dissolved in 85% phosphoric acid at room temperature and mixed with water as coagulant to prepare a supersaturated solution of cello-oligosaccharides, which was immediately applied to medical gauze (Figure 2a,b). It is noted that the phosphoric acid-catalyzed hydrolysis of cellulose is very slow at room temperature^26,27^ and negligible in this procedure. After 2 h for the self-assembly of cello-oligosaccharides (Figure 2c), the gauze was rinsed with water to remove phosphoric acid and unassembled oligosaccharides, freeze-dried, and observed by scanning electron microscopy (SEM). SEM images at low magnification revealed microfibrous structures (Figure 2d). The microfibrous structures were similar to those of the raw gauze materials and phosphoric acid-treated gauze without cello-oligosaccharides (Figure S2), indicating the maintenance of the gauze microfibrous structures even after the self-assembly of cello-oligosaccharides. High magnification images revealed that the surfaces of the microfibers with cello-oligosaccharides were entirely covered with nanospike structures (Figure 2e,f). The nanospikes were several tens of nanometers in diameter. In contrast, the gauze microfibers without cello-oligosaccharides had smooth surfaces (Figure S2). These results show that cello-oligosaccharides self-assembled into nanospike-shaped structures on gauze microfiber surfaces (Figure 1a).

**Figure 2:**
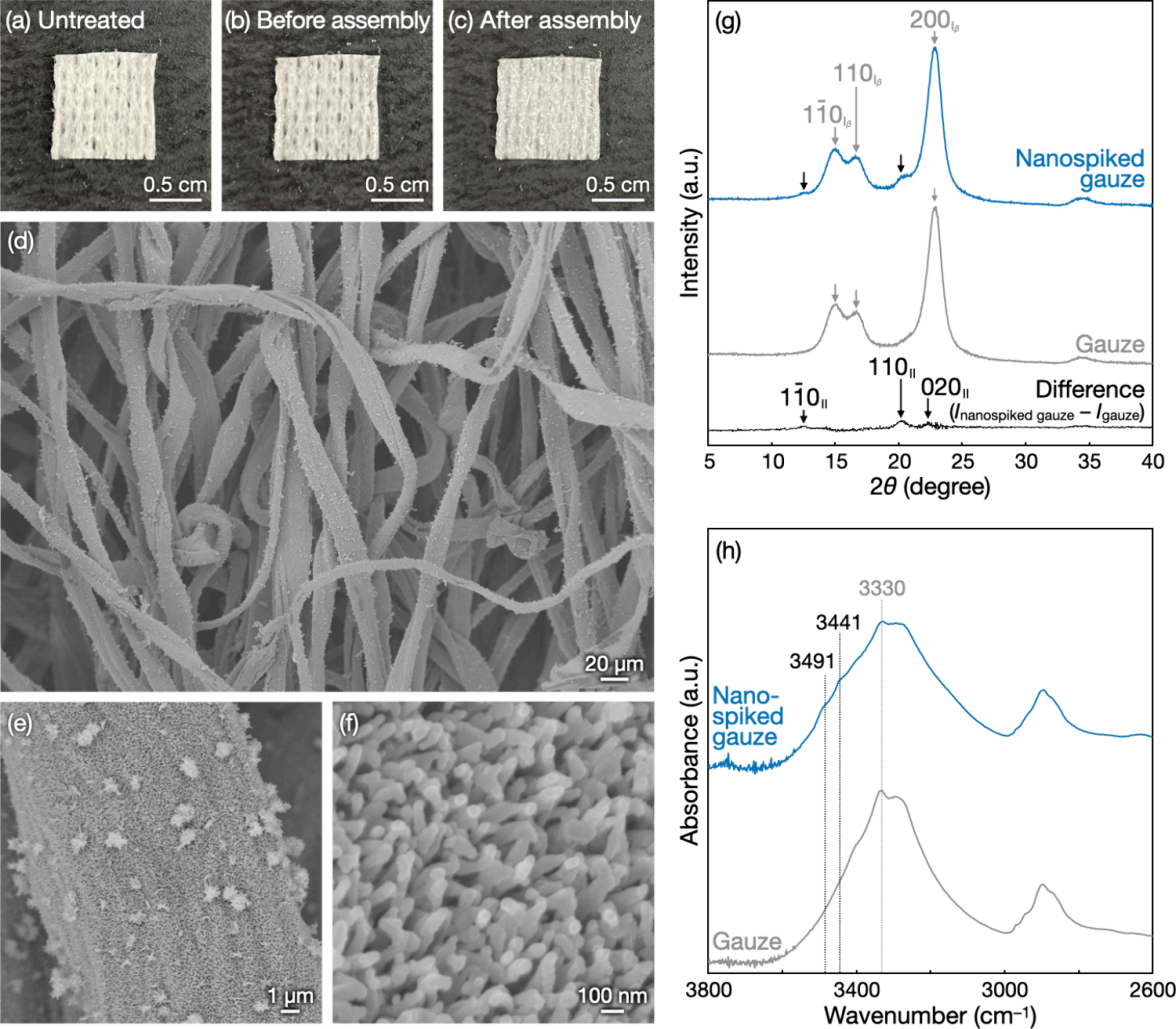
Fabrication of nanospiked gauze. Photographs of pieces of gauze (a) before and (b) after the addition of supersaturated solutions of cello-oligosaccharides and (c) after subsequent incubation at room temperature for self-assembly. SEM images of nanospiked gauze showing (d) multiple microfibers, (e) a single microfiber, and (f) a fiber surface with cello-oligosaccharide nanospikes. (g) XRD profiles of the nanospiked gauze and the raw gauze and their difference; intensities for the raw gauze (*I*_gauze_) was subtracted from those for the nanospiked gauze (*I*_nanospiked gauze_). (h) ATR-FTIR absorption spectra of the nanospiked gauze and the raw gauze.

The cello-oligosaccharides constituting nanospikes were in the cellulose II crystal allomorph (Figure 2g,h). The X-ray diffraction (XRD) profiles of the gauze with cello-oligosaccharide nanospikes showed major three peaks of the cellulose I_β_ allomorph, which is of the raw cotton gauze, and small additional peaks (Figure 2g).^29^ Subtraction of the XRD profile of the raw gauze clearly revealed that these small additional peaks were of the cellulose II allomorph. Moreover, attenuated total reflection-Fourier transform infrared (ATR-FTIR) absorption spectra of the nanospiked gauze showed a peak at 3330 cm^–1^ for the cellulose I allomorph and additional peaks at 3441 and 3491 cm^–1^, which were attributed to the intrachain hydrogen-bonded hydroxyl groups in the cellulose II allomorph (Figure 2h).^30^

Cellulose II is the most stable allomorph of cellulose and is common among in vitro cello-oligosaccharide assemblies, including nanospikes formed on filter paper.^25,31−34^ Given the difference in crystal allomorph between the cellulose gauze microfibers and the cello-oligosaccharide nanospikes, cello-oligosaccharides appeared to self-assemble into the most stable allomorph through heterogeneous nucleation on the microfiber surfaces, rather than epitaxial-like crystal growth. Such assembly pathways through heterogeneous nucleation have been found for the self-assembly of cello-oligosaccharides on filter paper^25,35^ and polymer nonwoven fabrics.^36^ Although it is currently unclear why cello-oligosaccharides adopted the nanospike morphology, substrate species for heterogeneous nucleation and solvents during self-assembly seem to be important factors.^35,36^

The heights of the nanospikes were controllable (Figure S3). Decreases in cello-oligosaccharide concentration from 1% (w/v) to 0.5, 0.25, and 0.1% (w/v) resulted in the formation of shorter nanospikes with decreasing the concentrations. XRD and ATR-FTIR analyses indicated that cello-oligosaccharides were in the cellulose II allomorph, irrespective of their concentration during self-assembly (Figure S4 and S5). Notably, the diameter of the nanospikes hardly varied, irrespective of cello-oligosaccharide concentrations (Figure S3). This implies that the nanospikes grew from the gauze microfiber surfaces while maintaining their diameter.

Bacterial adhesion to the nanospiked gauze was investigated (Figure 3). The nanospiked gauze was immersed in suspensions of *Escherichia coli* (*E. coli*) or *Pseudomonas aeruginosa* (*P. aeruginosa*) containing 0, 1, or 10% fetal bovine serum (FBS). After incubation for 24 h and subsequent washing with phosphate-buffered saline, the samples were observed by confocal laser scanning microscopy (CLSM) with SYTO 9–propidium iodide staining, where live and dead bacterial cells were colored green and red, respectively. *E. coli* cells adhered onto gauze microfibers were mostly observed as green dots, irrespective of the presence of nanospikes on microfibers or the presence of FBS in solutions, indicating that the cello-oligosaccharide nanospikes had negligible bactericidal effects under the conditions investigated (Figure 3a). At 0% FBS, the nanospiked gauze had fewer adhered *E. coli* cells than the raw gauze (Figure 3a, left). This indicates that the cello-oligosaccharide nanospikes have low bacterial adhesion properties in the absence of proteins (Figure 1b, left), probably because of the small contact area between the nanospiked surfaces and bacterial cells and the high hydration of the cello-oligosaccharide assembly surfaces.^37^

**Figure 3:**
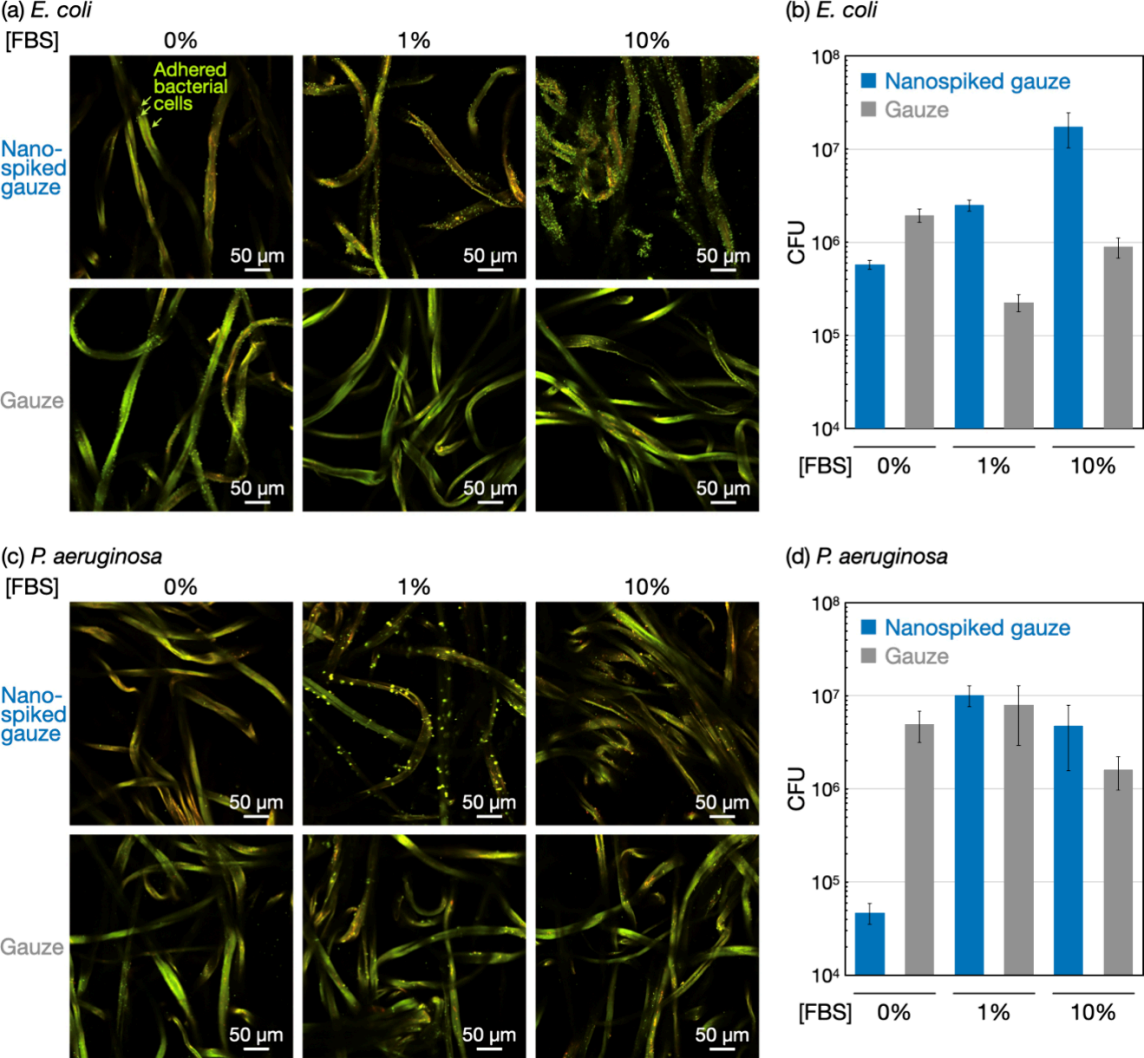
Bacterial adhesion to the nanospiked gauze in the absence and presence of FBS. (a, c) CLSM images and (b, d) colony counting assay results after incubation of the nanospiked gauze and the raw gauze for 24 h in suspensions of *E. coli* (a, b) or *P. aeruginosa* (c, d). The green fluorescence and the red fluorescence in CLSM images correspond to SYTO 9 and propidium iodide, respectively. For the colony counting assays, bacterial cells adhered to the nanospiked gauze or the raw gauze were corrected through sonication. The colony-forming unit (CFU) values are presented as the average of three individual trials, and the error bars represent the standard deviation of those trials.

The number of *E. coli* cells adhered onto the nanospiked gauze significantly increased with increasing FBS concentrations up to 10% FBS, while the number of *E. coli* cells adhered onto the raw gauze at 1% and 10% FBS was lower than that at 0% FBS (Figure 3a, middle and right). It is noted that *E. coli* grew in the presence of FBS (Figure S6), meaning that bacterial adhesion to the nanospiked gauze was mostly maintained even in the presence of FBS and that bacterial adhesion to the raw gauze was dramatically decreased by FBS. Quantification of the adhered bacterial cells by the colony counting assay yielded results consistent with the CLSM observation results (Figure 3b). In particular, it was shown that the nanospiked gauze had more than ten times larger amount of adhered *E. coli* cells than the raw gauze in the presence of 1% or 10% FBS. A decrease in incubation time from 24 to 1 h resulted in similar trends in *E. coli* cell adhesion, while the differences between the nanospiked gauze and the raw gauze were lower (Figures S7 and S8). The use of *P. aeruginosa* instead of *E. coli* provided similar trends in bacterial adhesion, although differences in the number of adhered bacterial cell between the nanospiked gauze and the raw gauze were less at 1% or 10% FBS (Figures 3c,d, S9, S10, and S11). These results demonstrate that the cello-oligosaccharide nanospikes promote bacterial adhesion in the presence of FBS. Such bacterial adhesion-promoting effects in the presence of proteins have rarely been observed for surface-nanospiked materials.^19,24^

It was suggested that adsorbed protein layers on the nanospikes were involved in the promoted bacterial adhesion (Figure 1b, right). The use of a protein, namely, bovine serum albumin (BSA), instead of FBS containing various proteins and other species led to similar results (Figures 4a,b, S12, S13, S14, and S15). For *E. coli*, the number of bacterial cells adhered to the nanospiked gauze was larger than that to the raw gauze at 1 or 10 mg mL^–1^ BSA (Figure 4a). For *P. aeruginosa*, the low bacterial adhesion properties of the nanospiked gauze diminished significantly with increasing BSA concentration (Figure 4b). It is mentioned that bacterial growth in BSA solutions was less than that in FBS solutions (Figures S13 and S15). These results show that not only various proteins and other species contained in FBS, but also a single species of protein can induce the bacterial adhesion-promoting effects of the nanospiked gauze. To gain insight into the effects of proteins, the nanospiked gauze was immersed in green fluorescent-labeled BSA solutions for 24 h. As a result, the nanospiked gauze microfibers were green-colored, indicating that the BSA molecules were adsorbed onto the microfiber surfaces to form adsorbed protein layers (Figure 4c, top). Notably, the microfibers of the raw gauze were slightly more colored in the fluorescent-labeled BSA solutions (Figure 4c, bottom), suggesting a larger amount of adsorbed proteins for the raw gauze. The antibiofouling properties of cello-oligosaccharide assemblies^38,39^ seemed to reduce the amount of protein adsorbed onto the nanospiked gauze. The adsorbed protein layers should play a role in bacterial adhesion, as discussed below.

**Figure 4:**
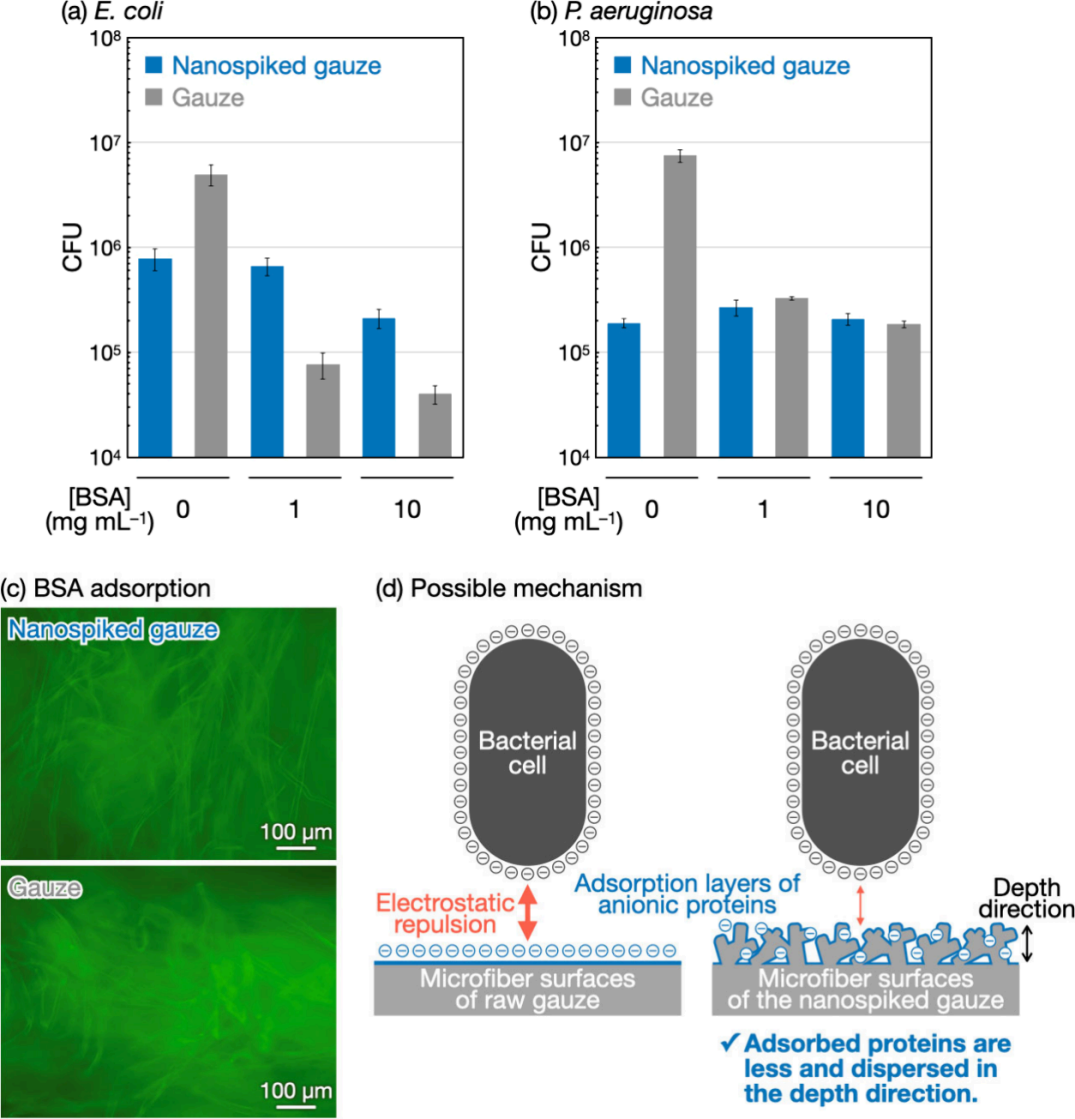
Mechanistic studies using BSA for bacterial adhesion behaviors on the nanospiked surfaces in the presence of proteins. Bacterial adhesion tests using (a) *E. coli* and (b) *P. aeruginosa* for the nanospiked gauze and the raw gauze in the presence of BSA by colony counting assay after 24 h of incubation. The CFU values are presented as the average of three individual trials, and the error bars represent the standard deviation of those trials. (c) Fluorescence microscopy images of the nanospiked gauze and the raw gauze after incubation for 24 h in fluorescent-labeled BSA solutions. (d) Schematic illustration for the possible mechanisms of the bacterial adhesion-promoting effects of the nanospiked gauze by comparing with the raw gauze.

The possible mechanisms of the bacterial adhesion-promoting effects of the nanospiked gauze in the presence of proteins are schematically shown in Figure 4d. Most proteins in the serum and BSA have net negative charges under physiological conditions.^40−42^ It was suggested that these proteins were adsorbed onto the microfibers of the raw gauze and the nanospiked gauze (Figure 4c). Consequently, the gauze microfiber surfaces with adsorbed protein layers appeared to have negative charges. For the raw gauze, the negatively charged surfaces contributed to preventing the adhesion of negatively charged *E. coli* and *P. aeruginosa* cells by electrostatic repulsion (Figure 4d, left). For the nanospiked gauze, the amount of adsorbed protein was less than that for the raw gauze (Figure 4c), meaning a less negative charge on the nanospiked surfaces. Moreover, the nanospike morphology dispersed the negatively charged proteins in the depth direction (Figure 4d, right). A previous calculation of the DLVO interactions between negatively charged bacteria and negatively charged rough surfaces using the surface element integration method indicated that increasing the surface roughness decreased the energy barrier for bacterial adhesion in a linear fashion.^43^ Consequently, the nanospiked surfaces should be more accessible to bacterial cells. These plausible mechanisms seem to promote the adhesion of negatively charged bacterial cells to the nanospiked surfaces in the presence of proteins.

Given its material properties (i.e., microfibrous and soft) and unique effects on bacteria (i.e., bacterial adhesion-promoting effects in the presence of proteins), the nanospiked gauze developed in this study has potential biomedical applications that are different from the previously considered applications of nanospiked materials (e.g., bactericidal implants).^5,6^ In fact, the nanospiked gauze was found to be useful for removing pathogenic bacteria from deep burn wounds (Figure 1c and 5). A full-thickness burn injury was made in mice according to a previously reported procedure (Figure 5b),^44^ inoculated with *P. aeruginosa*, and covered with the nanospiked gauze (Figure 5c). After 1 d, the nanospiked gauze was in a wet state with wound exudate (Figure 5d). The nanospiked gauze was removed from the deep burn wounds (Figure 5e), and the amount of *P. aeruginosa* in the wounds was quantified by colony counting assay using a selective medium for *Pseudomonas* species. The assay revealed that the bacterial load in deep burn wounds was successfully decreased by the nanospiked gauze when compared with the raw gauze (Figure 5f). This indicates that *P. aeruginosa* cells favorably adhered to the nanospiked surfaces in the presence of wound exudate containing proteins, thus reducing bacterial cells on the wounds (Figure 5a). Collectively, gauze decorated with cello-oligosaccharide nanospikes will be useful for reducing bacterial load in wounds with exudate containing proteins. The removal of bacteria via adhesion is attractive for infection control in wounds because removing bacteria without their death and disruption minimizes the release of endotoxins and other bacterial cell components causing inflammatory responses.^45^

**Figure 5:**
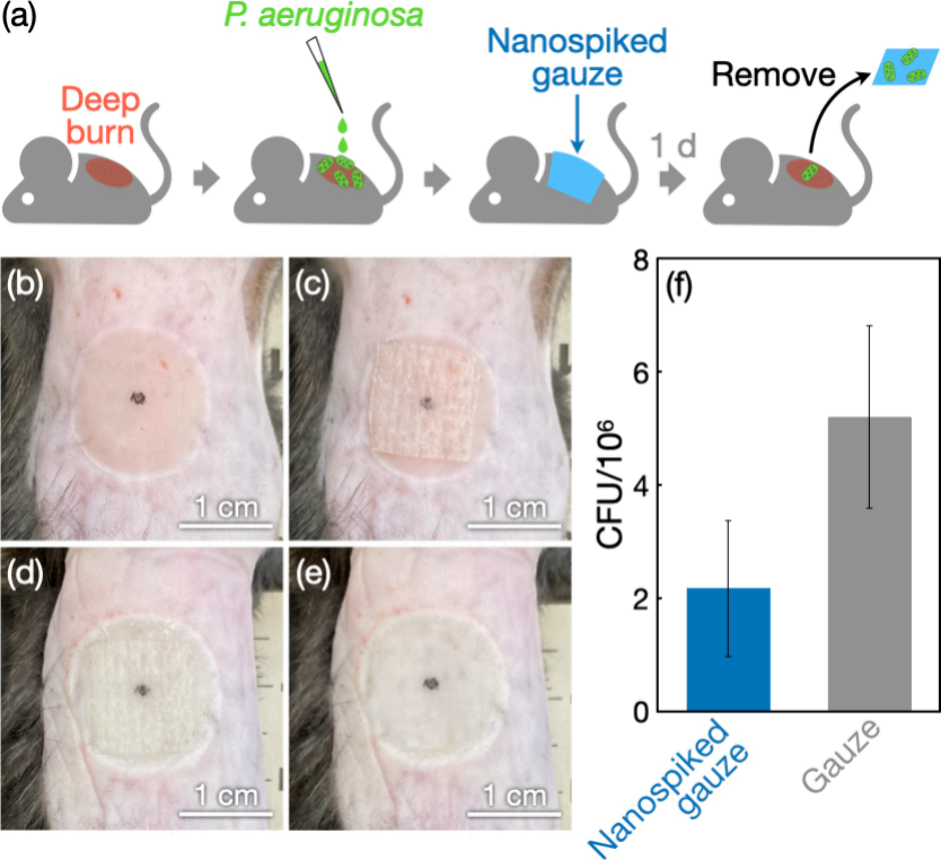
Removal of *P. aeruginosa* from deep burn wounds in mice using the nanospiked gauze. (a) Schematic outline of the experimental procedure. (b–e) Photographs of deep burn wounds on the dorsal skin of mice. Deep burn wounds were made on the dorsal skin of mice (b) and covered with the nanospiked gauze (c). After 1 d (d), the nanospiked gauze was removed from the wounds (e). (f) Colony counting assay results for *P. aeruginosa* from the deep burn wounds after the removal of the nanospiked gauze or the raw gauze. The CFU values are presented as the average of four individual trials, and the error bars represent the standard deviation of those trials.

In conclusion, we have demonstrated the decoration of gauze microfiber surfaces with nanospike structures via the self-assembly of cello-oligosaccharides, the bacterial adhesion-promoting effects of the cello-oligosaccharide nanospikes in the presence of proteins, and the use of the nanospiked gauze to reduce bacterial load in deep burn wounds. Cello-oligosaccharides self-assembled via heterogeneous nucleation into the cellulose II allomorph on gauze microfiber surfaces to form nanospike structures with diameters of several tens of nanometers. The resultant nanospiked gauze had low bacterial adhesion properties in the absence of proteins, whereas in the presence of FBS or BSA, bacterial cells adhered more to the nanospiked gauze than to the raw gauze. It was suggested that, for the cello-oligosaccharide nanospikes, adsorbed proteins with a net negative charge were less and dispersed in the depth direction because of the antibiofouling properties of cello-oligosaccharide assemblies and the nanospike morphology, respectively. Consequently, the energy barrier for bacterial adhesion appeared to decrease, promoting the adhesion of negatively charged bacterial cells to the nanospiked surfaces in the presence of proteins. Furthermore, the bacterial adhesion-promoting effects of the cello-oligosaccharide nanospikes in the presence of proteins were exploited to remove pathogenic bacteria from deep burn wounds with exudate containing proteins.

This study has two important findings. One is that the self-assembly of cello-oligosaccharides allows for the decoration of curved surfaces with nanospike structures in a bottom-up manner. Given that cello-oligosaccharide assembly through heterogeneous nucleation occurs not only on cellulose materials^25,35^ but also on synthetic polymers (e.g., polyolefin, polyester, and vinylon),^36^ this method can be applied to various medical materials to endow the conventional materials with infection control abilities. A significant advantage of nanostructured cello-oligosaccharide assemblies compared to other nanostructured organic materials is that the crystalline oligosaccharide assemblies, despite having hydrophilic surfaces, are highly stable under aqueous conditions.^32,46^ Therefore, materials with cello-oligosaccharide nanospikes will be robustly used in medicine. The other important finding is that the presence of proteins and other biomolecular species may alter the effects of nanospike structures on bacteria. This indicates that the antibacterial effects of nanospiked materials need to be investigated in the presence of proteins and other biomolecular species for future medical applications and that nanospike structures may have unique usability in vivo. In fact, the removal of bacteria from wounds via bacterial adhesion to the nanospiked gauze demonstrated in this study is attractive for infection control, as it will decrease bacterial load while minimizing bacterial cell death causing inflammatory responses. Collectively, this study contributes to the development of advanced nanostructured materials that can combat bacteria and other pathogens for infection control.

## Supporting Information Available

Detailed experimental procedures; mass spectrum of cello-oligosaccharides; SEM images, XRD profiles, and IR absorption spectra of gauze samples; CLSM images of gauze samples with adhered bacterial cells; colony counting assay for bacterial cells adhered to gauze samples (PDF)

## References

[ref1] Global Mortality Associated with 33 Bacterial Pathogens in 2019: A Systematic Analysis for the Global Burden of Disease Study 2019. Lancet 2022, 400 (10369), 2221–2248. 10.1016/S0140-6736(22)02185-7. 36423648 PMC9763654

[ref2] Global Burden of Bacterial Antimicrobial Resistance 1990–2021: A Systematic Analysis with Forecasts to 2050. Lancet 2024, 404 (10459), 1199–1226. 10.1016/S0140-6736(24)01867-1. 39299261 PMC11718157

[ref3] Anselme K.; Davidson P.; Popa A. M.; Giazzon M.; Liley M.; Ploux L. The Interaction of Cells and Bacteria with Surfaces Structured at the Nanometre Scale. Acta Biomater. 2010, 6 (10), 3824–3846. 10.1016/j.actbio.2010.04.001. 20371386

[ref4] Hasan J.; Chatterjee K. Recent Advances in Engineering Topography Mediated Antibacterial Surfaces. Nanoscale 2015, 7 (38), 15568–15575. 10.1039/C5NR04156B. 26372264 PMC4642214

[ref5] Linklater D. P.; Baulin V. A.; Juodkazis S.; Crawford R. J.; Stoodley P.; Ivanova E. P. Mechano-Bactericidal Actions of Nanostructured Surfaces. Nat. Rev. Microbiol. 2021, 19 (1), 8–22. 10.1038/s41579-020-0414-z. 32807981

[ref6] Hawi S.; Goel S.; Kumar V.; Pearce O.; Ayre W. N.; Ivanova E. P. Critical Review of Nanopillar-Based Mechanobactericidal Systems. ACS Appl. Nano Mater. 2022, 5 (1), 1–17. 10.1021/acsanm.1c03045.

[ref7] Mohanan S.; Sathish C. I.; Adams T. J.; Kan S.; Liang M.; Vinu A. A Dual Protective Drug Delivery System Based on Lipid Coated Core-Shell Mesoporous Silica for Efficient Delivery of Cabazitaxel to Prostate Cancer Cells. Bull. Chem. Soc. Jpn. 2023, 96 (10), 1188–1195. 10.1246/bcsj.20230167.

[ref8] Song J.; Kawakami K.; Ariga K. Nanoarchitectonics in Combat against Bacterial Infection Using Molecular, Interfacial, and Material Tools. Curr. Opin. Colloid Interface Sci. 2023, 65, 101702 10.1016/j.cocis.2023.101702.

[ref9] Nishimura S.; Tanaka M. The Intermediate Water Concept for Pioneering Polymeric Biomaterials: A Review and Update. Bull. Chem. Soc. Jpn. 2023, 96 (9), 1052–1070. 10.1246/bcsj.20230168.

[ref10] Xu L.-C.; Siedlecki C. A. Submicron-Textured Biomaterial Surface Reduces Staphylococcal Bacterial Adhesion and Biofilm Formation. Acta Biomater. 2012, 8 (1), 72–81. 10.1016/j.actbio.2011.08.009. 21884831

[ref11] Xu L.-C.; Siedlecki C. A. *Staphylococcus epidermidis* Adhesion on Hydrophobic and Hydrophilic Textured Biomaterial Surfaces. Biomed. Mater. 2014, 9 (3), 035003 10.1088/1748-6041/9/3/035003. 24687453

[ref12] Ivanova E. P.; Hasan J.; Webb H. K.; Truong V. K.; Watson G. S.; Watson J. A.; Baulin V. A.; Pogodin S.; Wang J. Y.; Tobin M. J.; Löbbe C.; Crawford R. J. Natural Bactericidal Surfaces: Mechanical Rupture of *Pseudomonas aeruginosa* Cells by Cicada Wings. Small 2012, 8 (16), 2489–2494. 10.1002/smll.201200528. 22674670

[ref13] Ivanova E. P.; Hasan J.; Webb H. K.; Gervinskas G.; Juodkazis S.; Truong V. K.; Wu A. H. F.; Lamb R. N.; Baulin V. A.; Watson G. S.; Watson J. A.; Mainwaring D. E.; Crawford R. J. Bactericidal Activity of Black Silicon. Nat. Commun. 2013, 4 (1), 2838 10.1038/ncomms3838. 24281410 PMC3868328

[ref14] Zhao S.; Li Z.; Linklater D. P.; Han L.; Jin P.; Wen L.; Chen C.; Xing D.; Ren N.; Sun K.; Juodkazis S.; Ivanova E. P.; Jiang L. Programmed Death of Injured *Pseudomonas aeruginosa* on Mechano-Bactericidal Surfaces. Nano Lett. 2022, 22 (3), 1129–1137. 10.1021/acs.nanolett.1c04243. 35040647

[ref15] Li Y.; Zhu B.; Li Y.; Leow W. R.; Goh R.; Ma B.; Fong E.; Tang M.; Chen X. A Synergistic Capture Strategy for Enhanced Detection and Elimination of Bacteria. Angew. Chem., Int. Ed. 2014, 53 (23), 5837–5841. 10.1002/anie.201310135. 24648144

[ref16] Nakade K.; Jindai K.; Sagawa T.; Kojima H.; Shimizu T.; Shingubara S.; Ito T. Adhesion and Bactericidal Properties of a Wettability-Controlled Artificial Nanostructure. ACS Appl. Nano Mater. 2018, 1 (10), 5736–5741. 10.1021/acsanm.8b01340.

[ref17] Jindai K.; Nakade K.; Masuda K.; Sagawa T.; Kojima H.; Shimizu T.; Shingubara S.; Ito T. Adhesion and Bactericidal Properties of Nanostructured Surfaces Dependent on Bacterial Motility. RSC Adv. 2020, 10 (10), 5673–5680. 10.1039/C9RA08282D. 35497460 PMC9049231

[ref18] Jenkins J.; Mantell J.; Neal C.; Gholinia A.; Verkade P.; Nobbs A. H.; Su B. Antibacterial Effects of Nanopillar Surfaces Are Mediated by Cell Impedance, Penetration and Induction of Oxidative Stress. Nat. Commun. 2020, 11 (1), 1626 10.1038/s41467-020-15471-x. 32242015 PMC7118135

[ref19] Visalakshan R. M.; Bright R.; Burzava A. L. S.; Barker A. J.; Simon J.; Ninan N.; Palms D.; Wood J.; Martínez-Negro M.; Morsbach S.; Mailänder V.; Anderson P. H.; Brown T.; Barker D.; Landfester K.; Vasilev K. Antibacterial Nanostructured Surfaces Modulate Protein Adsorption, Inflammatory Responses, and Fibrous Capsule Formation. ACS Appl. Mater. Interfaces 2023, 15 (1), 220–235. 10.1021/acsami.2c13415. 36416784

[ref20] Serrano C.; García-Fernández L.; Fernández-Blázquez J. P.; Barbeck M.; Ghanaati S.; Unger R.; Kirkpatrick J.; Arzt E.; Funk L.; Turón P.; del Campo A. Nanostructured Medical Sutures with Antibacterial Properties. Biomaterials 2015, 52, 291–300. 10.1016/j.biomaterials.2015.02.039. 25818435

[ref21] Hsiao S.-W.; Venault A.; Yang H.-S.; Chang Y. Bacterial Resistance of Self-Assembled Surfaces Using PPO_*m*_-*b*-PSBMA_n_ Zwitterionic Copolymer - Concomitant Effects of Surface Topography and Surface Chemistry on Attachment of Live Bacteria. Colloids Surf. B Biointerfaces 2014, 118, 254–260. 10.1016/j.colsurfb.2014.03.051. 24794801

[ref22] Heckmann T. S.; Schiffman J. D. Spatially Organized Nanopillar Arrays Dissimilarly Affect the Antifouling and Antibacterial Activities of *Escherichia coli* and *Staphylococcus aureus*. ACS Appl. Nano Mater. 2020, 3 (2), 977–984. 10.1021/acsanm.9b01942.

[ref23] Fujimoto K.; Saito A.; Kotsuchibashi Y. Cicada-Wing-Inspired Nanopillar Hydrogels Consisting of Poly(vinyl alcohol) and Poly(methacrylic acid) for Capturing Bacteria through Their Flexibility and Wide Range of Motion. ACS Macro Lett. 2022, 11 (6), 727–732. 10.1021/acsmacrolett.2c00126. 35579174

[ref24] Martins de Sousa K.; Linklater D. P.; Baulin V. A.; Dekiwadia C.; Mayes E.; Murdoch B. J.; Le P. H.; Fluke C. J.; Boshkovikj V.; Wen C.; Crawford R. J.; Ivanova E. P. Understanding the Influence of Serum Proteins Adsorption on the Mechano-bactericidal Efficacy and Immunomodulation of Nanostructured Titanium. Adv. Mater. Interfaces 2024, 11 (17), 2301021 10.1002/admi.202301021.

[ref25] Hata Y.; Hiruma S.; Sakurai Y.; Sugiura K.; Miyazaki H.; Serizawa T.; Nakamura S. Nanospiked Paper: Microfibrous Cellulose Materials Nanostructured via Partial Hydrolysis and Self-Assembly. Carbohydr. Polym. 2023, 300, 120257 10.1016/j.carbpol.2022.120257. 36372485

[ref26] Isogai A.; Usuda M. Preparation of Low-Molecular Weight Celluloses Using Phosphoric Acid. Mokuzai Gakkaishi 1991, 37 (4), 339–344.

[ref27] Wei S.; Kumar V.; Banker G. S. Phosphoric Acid Mediated Depolymerization and Decrystallization of Cellulose: Preparation of Low Crystallinity Cellulose — A New Pharmaceutical Excipient. Int. J. Pharm. 1996, 142 (2), 175–181. 10.1016/0378-5173(96)04673-X.

[ref28] Isobe N.; Ono Y.; Nishiyama Y.; Roux D.; Isogai A. Quantitative Analysis of the Formation of Monodisperse Cello-oligomers Obtained by Phosphoric Acid Hydrolysis. Cellulose 2023, 30 (13), 8235–8243. 10.1007/s10570-023-05415-1.

[ref29] French A. D. Idealized Powder Diffraction Patterns for Cellulose Polymorphs. Cellulose 2014, 21 (2), 885–896. 10.1007/s10570-013-0030-4.

[ref30] Hishikawa Y.; Togawa E.; Kondo T. Characterization of Individual Hydrogen Bonds in Crystalline Regenerated Cellulose Using Resolved Polarized FTIR Spectra. ACS Omega 2017, 2 (4), 1469–1476. 10.1021/acsomega.6b00364. 31457518 PMC6640953

[ref31] Klemm D.; Heublein B.; Fink H.-P.; Bohn A. Cellulose: Fascinating Biopolymer and Sustainable Raw Material. Angew. Chem., Int. Ed. 2005, 44 (22), 3358–3393. 10.1002/anie.200460587. 15861454

[ref32] Hata Y.; Serizawa T. Self-Assembly of Cellulose for Creating Green Materials with Tailor-Made Nanostructures. J. Mater. Chem. B 2021, 9 (19), 3944–3966. 10.1039/D1TB00339A. 33908581

[ref33] Hata Y.; Serizawa T. Robust Gels Composed of Self-Assembled Cello-oligosaccharide Networks. Bull. Chem. Soc. Jpn. 2021, 94 (9), 2279–2289. 10.1246/bcsj.20210234.

[ref34] Zhong C.; Nidetzky B. Bottom-Up Synthesized Glucan Materials: Opportunities from Applied Biocatalysis. Adv. Mater. 2024, 36, 2400436 10.1002/adma.202400436. 38514194

[ref35] Hanamura M.; Sawada T.; Serizawa T. In-Paper Self-Assembly of Cellulose Oligomers for the Preparation of All-Cellulose Functional Paper. ACS Sustainable Chem. Eng. 2021, 9 (16), 5684–5692. 10.1021/acssuschemeng.1c00815.

[ref36] Mizuuchi Y.; Hata Y.; Sawada T.; Serizawa T. Surface-Mediated Self-Assembly of Click-Reactive Cello-oligosaccharides for Fabricating Functional Nonwoven Fabrics. Sci. Technol. Adv. Mater. 2024, 25 (1), 2311052 10.1080/14686996.2024.2311052. 38361530 PMC10868462

[ref37] Serizawa T.; Yamaguchi S.; Amitani M.; Ishii S.; Tsuyuki H.; Tanaka Y.; Sawada T.; Kawamura I.; Watanabe G.; Tanaka M. Alkyl Chain Length-Dependent Protein Nonadsorption and Adsorption Properties of Crystalline Alkyl β-Celluloside Assemblies. Colloids Surf. B Biointerfaces 2022, 220, 112898 10.1016/j.colsurfb.2022.112898. 36244130

[ref38] Sugiura K.; Sawada T.; Hata Y.; Tanaka H.; Serizawa T. Distinguishing Anti-PEG Antibodies by Specificity for the PEG Terminus Using Nanoarchitectonics-Based Antibiofouling Cello-oligosaccharide Platforms. J. Mater. Chem. B 2024, 12 (3), 650–657. 10.1039/D3TB01723K. 38088066

[ref39] Suehiro F.; Hata Y.; Sawada T.; Serizawa T. Freeze-Dryable, Stable, and Click-Reactive Nanoparticles Composed of Cello-oligosaccharides for Biomolecular Sensing. ACS Appl. Bio Mater. 2024, 7 (6), 4007–4016. 10.1021/acsabm.4c00359. 38739554

[ref40] Busher J. T. Serum Albumin and Globulin. In Clinical methods: the history, physical, and laboratory examinations. 3rd ed.; Walker H. K., Hall W. D., Hurst J. W., Eds.; Butterworths: Boston, 1990; pp 497–499. 21250045

[ref41] Jin Y.; Luo G.; Oka T.; Manabe T. Estimation of Isoelectric Points of Human Plasma Proteins Employing Capillary Isoelectric Focusing and Peptide Isoelectric Point Markers. Electrophoresis 2002, 23 (19), 3385–3391. 10.1002/1522-2683(200210)23:19<3385::AID-ELPS3385>3.0.CO;2-H. 12373767

[ref42] Salis A.; Boström M.; Medda L.; Cugia F.; Barse B.; Parsons D. F.; Ninham B. W.; Monduzzi M. Measurements and Theoretical Interpretation of Points of Zero Charge/Potential of BSA Protein. Langmuir 2011, 27 (18), 11597–11604. 10.1021/la2024605. 21834579

[ref43] Mu M.; Liu S.; DeFlorio W.; Hao L.; Wang X.; Salazar K. S.; Taylor M.; Castillo A.; Cisneros-Zevallos L.; Oh J. K.; Min Y.; Akbulut M. Influence of Surface Roughness, Nanostructure, and Wetting on Bacterial Adhesion. Langmuir 2023, 39 (15), 5426–5439. 10.1021/acs.langmuir.3c00091. 37014907 PMC10848269

[ref44] Saito A.; Miyazaki H.; Fujie T.; Ohtsubo S.; Kinoshita M.; Saitoh D.; Takeoka S. Therapeutic Efficacy of an Antibiotic-Loaded Nanosheet in a Murine Burn-Wound Infection Model. Acta Biomater. 2012, 8 (8), 2932–2940. 10.1016/j.actbio.2012.04.019. 22525350

[ref45] Cooper R. The Contribution of Microbial Virulence to Wound Infection. Br. J. Community Nurs. 2002, 7 (Sup4), 10–14. 10.12968/bjcn.2002.7.Sup4.12615. 12066077

[ref46] Hata Y.; Serizawa T. Nanoarchitectonics of Cello-oligosaccharides: A Route toward Artificial Nanocelluloses. Adv. Colloid Interface Sci. 2025, 336, 103361 10.1016/j.cis.2024.103361. 39642432

