# Supplementary material for: Nanospiked Cellulose Gauze That Attracts Bacteria with Biomolecules for Reducing Bacterial Load in Burn Wounds
Source: Nano Lett. 2025 Jan 13;25(3):1177–84. doi: 10.1021/acs.nanolett.4c05773 (PMC11760149; doi:10.1021/acs.nanolett.4c05773)
Supplement: Supplementary file 1 — nl4c05773_si_001.pdf [file nl4c05773_si_001.pdf]

Supporting Information

# Nanospiked Cellulose Gauze That Attracts Bacteria with Biomolecules for Reducing Bacterial Load in Burn Wounds

*Yuuki Hata,*[†,‡] Hiromi Miyazaki,[‡] Sayaka Okamoto,[†] Takeshi Serizawa,[†] and Shingo Nakamura[‡]*

[†]Department of Chemical Science and Engineering, School of Materials and Chemical Technology, Institute of Science Tokyo, 2-12-1-H-121 Ookayama, Meguro-ku, Tokyo 152-8550, Japan.

[‡]Division of Biomedical Engineering, National Defense Medical College Research Institute, 3-2 Namiki, Tokorozawa-shi, Saitama 359-8513, Japan

* Correspondence: hata@mct.isct.ac.jp (Y.H.)

## Materials and Methods

**Materials.** Nonwoven fabric gauze (Cross Gauze Cotton, cotton 100%) and saline were purchased from Osaki Medical (Aichi, Japan) and Otsuka Pharmaceutical Factory (Tokushima, Japan), respectively. Fetal bovine serum (FBS, qualified, Brazil, Gibco) and LIVE/DEAD® BacLight Bacterial Viability Kit (L13152) were purchased from Thermo Fisher Scientific (Massachusetts, USA). Phosphoric acid (85%) and Dulbecco's phosphate-buffered saline without calcium or magnesium (D-PBS(–)) were purchased from Wako Pure Chemical Industries (Osaka, Japan). Brain heart infusion broth and nutrient agar Nissui were purchased from Nissui Pharmaceutical (Tokyo, Japan). Avicel PH-101, 1% trifluoroacetic acid, acetonitrile, 2,5-dihydroxybenzoic acid, bovine serum albumin (BSA), and an albumin–fluorescein isothiocyanate conjugate (≥7 mol fluorescein isothiocyanate/mol albumin) were purchased from Merck (Darmstadt, Germany). *Escherichia coli* (*E. coli*, ATCC 51813) and *Pseudomonas aeruginosa* (*P. aeruginosa*, ATCC 27853) were purchased from Microbiologics (Minnesota, USA). Male C57BL/6J mice were purchased from Oriental Yeast (Tokyo, Japan). Ultrapure water with a resistivity greater than 18.2 MΩ cm at 25 °C was supplied by an RFU464TA instrument (Advantec, Tokyo, Japan) and used in all experiments.

**Preparation of Cello-oligosaccharides.** Microcrystalline cellulose (Avicel PH-101, 150 mg) in a powder state was added to 5 mL of 85% phosphoric acid while vortexing and then left to stand at room temperature for a few minutes to prepare a 3% (w/v) cellulose solutions. The cellulose solutions were incubated at 45 °C for 20 h for the hydrolysis of cellulose. To the resultant cello-oligosaccharide solutions (5 mL), 5 mL of ultrapure water was added as coagulant before keeping the mixtures at room temperature for 2 h. Precipitated cello-oligosaccharides were purified with ultrapure water through at least six centrifugation–redispersion cycles. For nanospiked gauze preparation, the cello-oligosaccharides were freeze-dried and stored at room temperature until use.

For matrix-assisted laser desorption/ionization time-of-flight (MALDI-TOF) mass spectrometry, never-dried cello-oligosaccharides were mixed with 2,5-dihydroxybenzoic acid, trifluoroacetic acid, and acetonitrile at concentrations of 2.0 mg $mL^{-1}$, 0.1% (v/v), and 50% (v/v), respectively, and deposited onto an AXIMA 384-well plate. After drying under ambient conditions, the samples were introduced into an AXIMA performance instrument (Shimadzu, Kyoto, Japan) equipped with a nitrogen laser ($\lambda$ = 337 nm) and pulsed ion extraction. Measurements were performed at an accelerating potential of 20 kV in the linear positive ion mode.

**Nanostructuring of Gauze.** The nonwoven fabric gauze was cut into pieces (1 cm × 1 cm). The freeze-dried cello-oligosaccharides were dissolved typically at 2% (w/v) in 85% phosphoric acid. Thirty microliters of the cello-oligosaccharide solutions was mixed with 30 μL of ultrapure water as coagulant. The resultant cello-oligosaccharide supersaturated solutions (typically 1% (w/v)) were immediately applied to a piece of the 1 cm × 1 cm gauze, which was left to stand at room temperature for 2 h to allow cello-oligosaccharides to self-assemble. The resultant nanospiked gauze was purified by immersion in ultrapure water for at least 2 d, during which ultrapure water was exchanged five times.

**Characterization of Nanospiked Gauze.** For X-ray diffraction (XRD) analysis and attenuated total reflection-Fourier transform infrared (ATR-FTIR) absorption spectroscopy, the purified nanospiked gauze containing water was frozen in liquid nitrogen and freeze-dried. XRD measurements were carried out using a MiniFlex600-C instrument (Rigaku, Tokyo, Japan) equipped with Cu K$\alpha$ radiation ($\lambda$ = 1.54 Å) and a HyPix-400 MF detector over a 2$\theta$ range of 5–40° ($\theta$ is the Bragg angle) with a step of 0.02° at a scan speed of 2° $min^{-1}$ under ambient conditions. FTIR measurements were carried out using an IRSpirit instrument (Shimadzu, Kyoto, Japan) equipped with an ATR unit at a cumulative measurement number of 100 and a resolution of 2.0 $cm^{-1}$ under ambient conditions.

For scanning electron microscopy (SEM), the purified nanospiked gauze containing water was immersed in 25, 50, and 75% ethanol, ethanol, ethanol–*tert*-butyl alcohol (1:1, v/v), and then *tert*-butyl alcohol. The resultant samples containing *tert*-butyl alcohol were frozen in liquid nitrogen and freeze-dried. The dried samples were fixed on the substrates using conductive carbon double-sided tape before being coated with osmium. A JSM-6340F instrument (JEOL, Tokyo, Japan) was operated at an accelerating voltage of 5 kV.

**Bacterial Adhesion Study.** *E. coli* or *P. aeruginosa* was added at $1 \times 10^7$ colony-forming units (CFU) $mL^{-1}$ to D-PBS(–) containing 0, 1, or 10% FBS or 1 or 10 mg $mL^{-1}$ BSA. 3 mL of the bacteria suspensions were added to a sterilized Petri dish (~30 mm in diameter), followed by immersion of a piece of the nanospiked gauze or the raw gauze and subsequent incubation at 37 °C for 1 or 24 h. The resultant gauze with bacterial cells was, for washing out unadhered bacterial cells, transferred into 8 mL of D-PBS(–) in a sterilized Petri dish (~60 mm in diameter) and gently stirred using a Wave-SI shaker (Taitec, Saitama, Japan) at a speed of 40 for 30 min. The samples were transferred into fresh D-PBS(–) and gently stirred for 30 min again.

For confocal laser scanning microscopy (CLSM), the washed nanospiked gauze or raw gauze with adhered bacterial cells was placed on a glass slide. Twenty microliters of aqueous solutions containing 12 μM SYTO 9 and 60 μM propidium iodide was added to the sample on a glass slide before covering the sample with a cover glass. An AX R instrument (Nikon, Tokyo, Japan) equipped with a Plan Apo VC 20x DIC N2 objective (NA 0.75) was operated with a 488 nm laser for excitation of both SYTO 9 and propidium iodide. The fluorescence of SYTO 9 and propidium iodide was detected at wavelength ranges of 500–550 nm and 587–684 nm, respectively, and merged to generate an image.

For colony counting assay, the washed nanospiked gauze or raw gauze with adhered bacterial cells was immersed in 1.5 mL of D-PBS(–) and sonicated for 5 min using a bath-type ultrasonic

cleaner (LEO-80, Tokyo Garasu Kikai, Tokyo, Japan) to liberate the bacterial cells from gauze surfaces. The resultant bacterial suspensions were appropriately diluted and added to Compact Dry CF and Compact Dry TC (Nissui Pharmaceutical, Tokyo, Japan) for *E. coli* and *P. aeruginosa*, respectively. The Compact Dry CF and Compact Dry TC were incubated at 35 °C for 1 d and 2 d, respectively, before colony counting.

**Protein Adsorption Study.** The nanospiked gauze and raw gauze were immersed in D-PBS(–) for more than 12 h. The gauze sample was transferred into 1 mL of 1 mg $mL^{-1}$ albumin–fluorescein isothiocyanate conjugate in D-PBS(–) and incubated for 24 h. After removing excess albumin–fluorescein isothiocyanate conjugate solutions, the samples were observed using a ZOE Fluorescent Cell Imager (Bio-Rad, California, USA) with a green channel (excitation: 480/17 nm, emission: 517/23 nm) at a gain of 7, an exposure time of 500 ms, an LED intensity of 1, and a contrast of 15.

**In Vivo Bacteria Removal Test.** All procedures were conducted according to the protocols approved by the National Defense Medical College Animal Care and Use Committee (permission number: 22035) and performed at the National Defense Medical College. Male C57BL/6J mice (8–9 weeks old, 21–24 g) were used. The mice were housed under standard laboratory conditions with a 12 hours light–dark cycle and a room temperature of 23 °C and had ad libitum access to water and food.

A full-thickness burn injury (deep burn) was made as previously described.[1] The surgical procedures were performed for mice under anesthesia with isoflurane. The dorsal hair was clipped and depilated using a depilatory agent. The dorsal skin area (15 mm in diameter) was exposed to water at 90 °C for 7 s using a Walker-Mason template to induce a full-thickness burn-injury. To prevent dehydration, 1 mL of saline was intraperitoneally administered to all mice. The wound site was inoculated with *P. aeruginosa* (10 µL, $1.8 \times 10^8$ CFU $mL^{-1}$), covered with the nanospiked gauze or raw gauze (1 cm × 1 cm), and wrapped with a semipermeable adhesive film (Opsite, Smith &

Nephew, Watford, UK) to protect the wound and prevent desiccation. After 1 d, the nanospiked gauze or raw gauze and the film were removed from the wound. A part of the wound (8 mm in diameter) was corrected using a biopsy punch and surgical scissors and homogenized in 1 mL of saline using a Physcotron NS-360D equipped with NS-3 (Microtec, Chiba, Japan) at 30 krpm. The resultant samples were appropriately diluted and added to Compact Dry PA (Nissui Pharmaceutical, Tokyo, Japan), a selective medium for *Pseudomonas* species, before incubation at 35 °C for 2 d and subsequent colony counting.

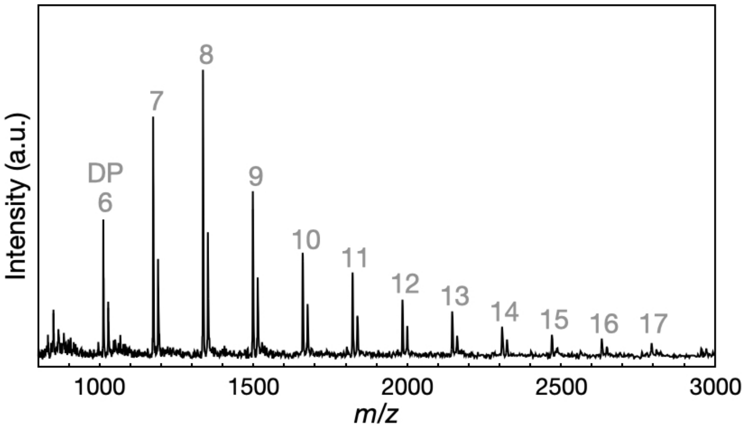


**Figure S1.** MALDI-TOF mass spectrum of cello-oligosaccharides used for the nanostructuring of gauze. The numbers above the peaks denote the degree of polymerization (DP) values of the oligosaccharides.

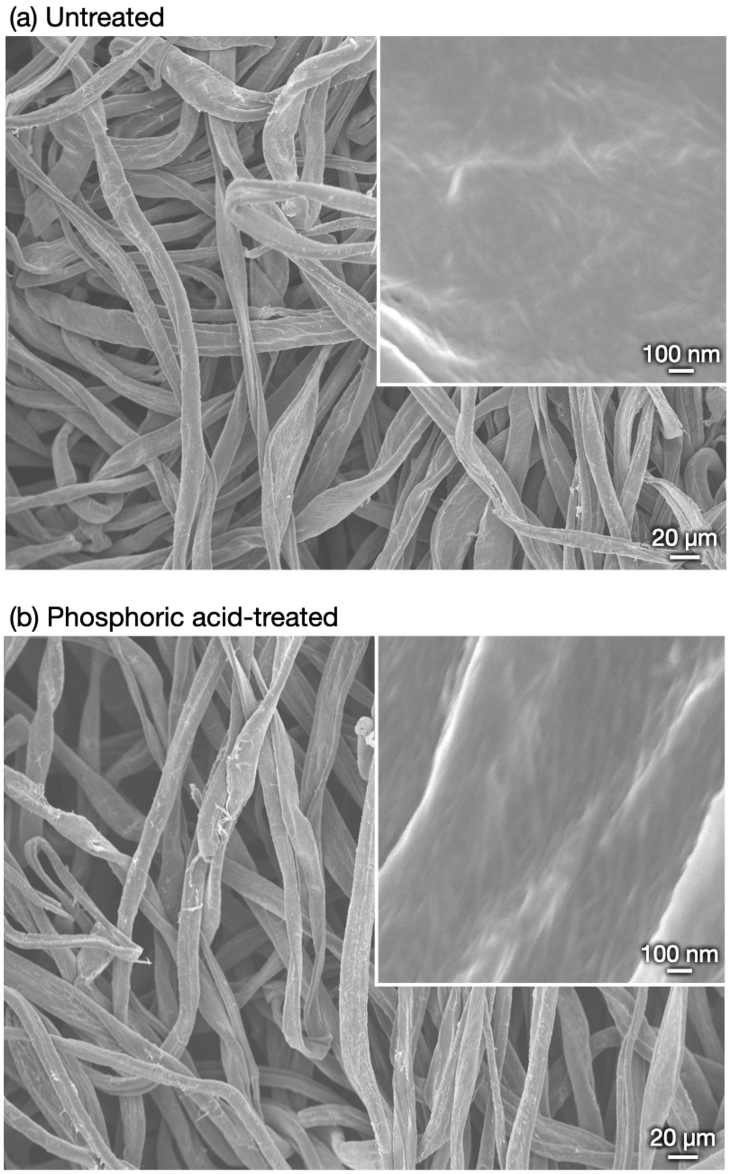


**Figure S2.** SEM images of (a) untreated (raw) gauze and (b) gauze treated with ~43% phosphoric acid (the solvent for cello-oligosaccharide assembly).

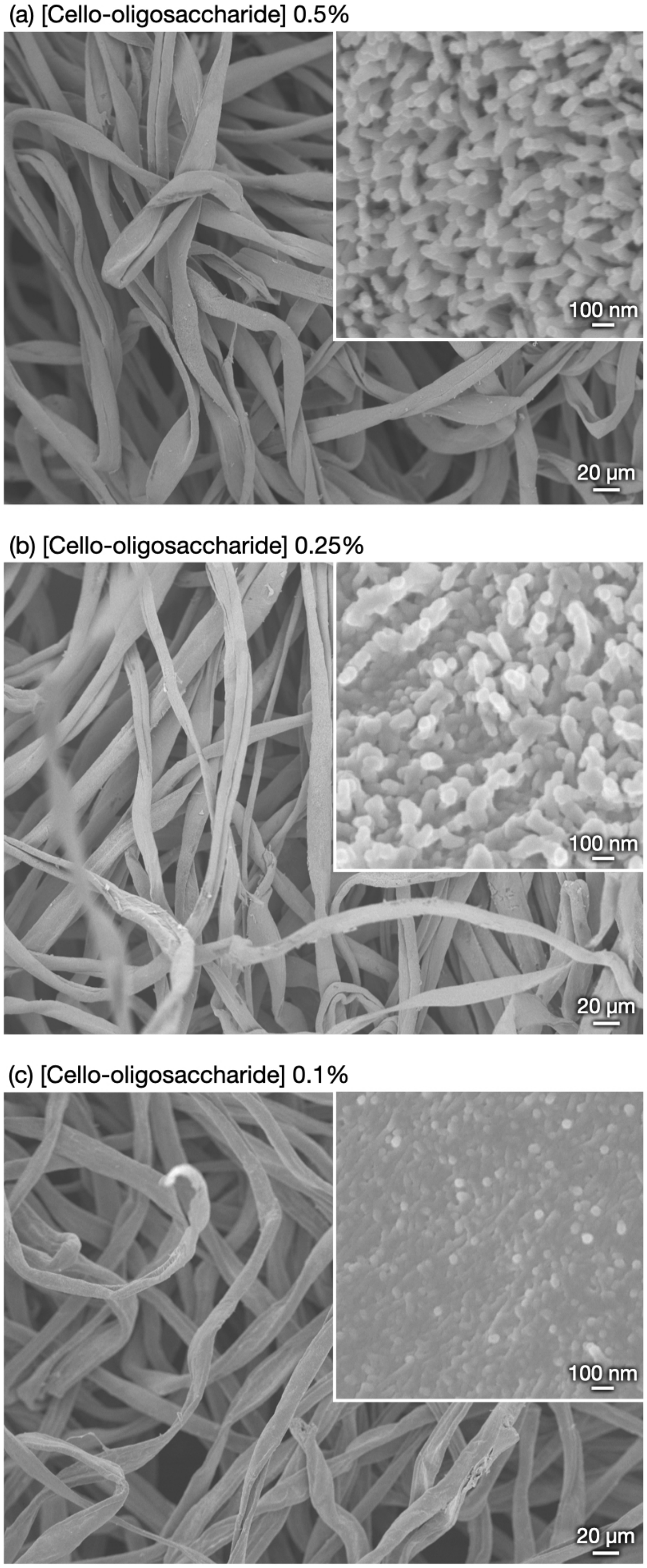


**Figure S3.** SEM images of gauze with cello-oligosaccharides assembled at (a) 0.5, (b) 0.25, and (c) 0.1%.

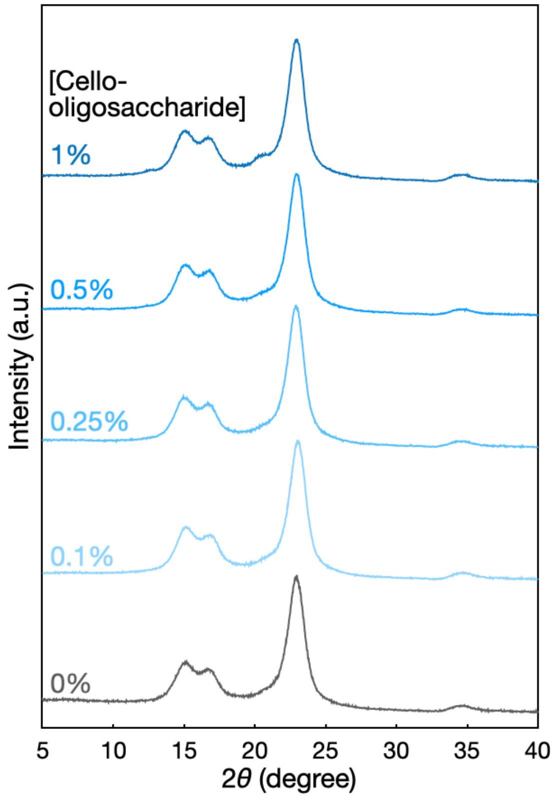


**Figure S4.** XRD profiles of gauze with cello-oligosaccharides assembled at different concentrations. The sample of "0%" is the gauze treated with ~43% phosphoric acid (the solvent for cello-oligosaccharide assembly).

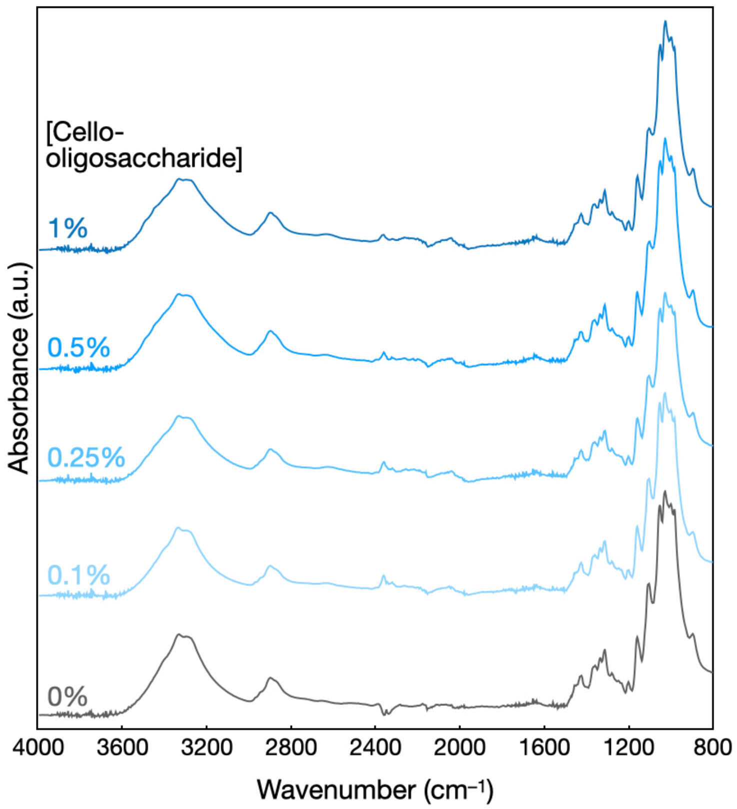


**Figure S5.** ATR-FTIR absorption spectra of gauze with cello-oligosaccharides assembled at different concentrations. The sample of "0%" is the gauze treated with ~43% phosphoric acid (the solvent for cello-oligosaccharide assembly).

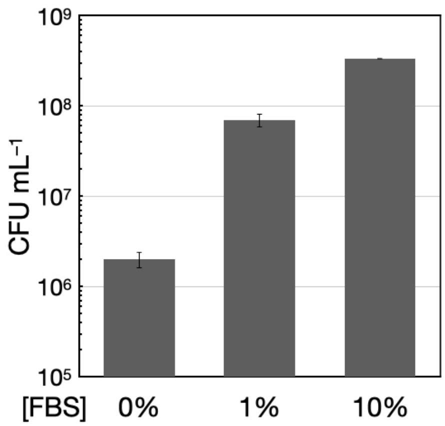


**Figure S6.** Concentrations of viable *E. coli* cells after incubation for 24 h in FBS solutions. The *E. coli* concentration before incubation was $1 \times 10^7$ CFU mL$^{-1}$.

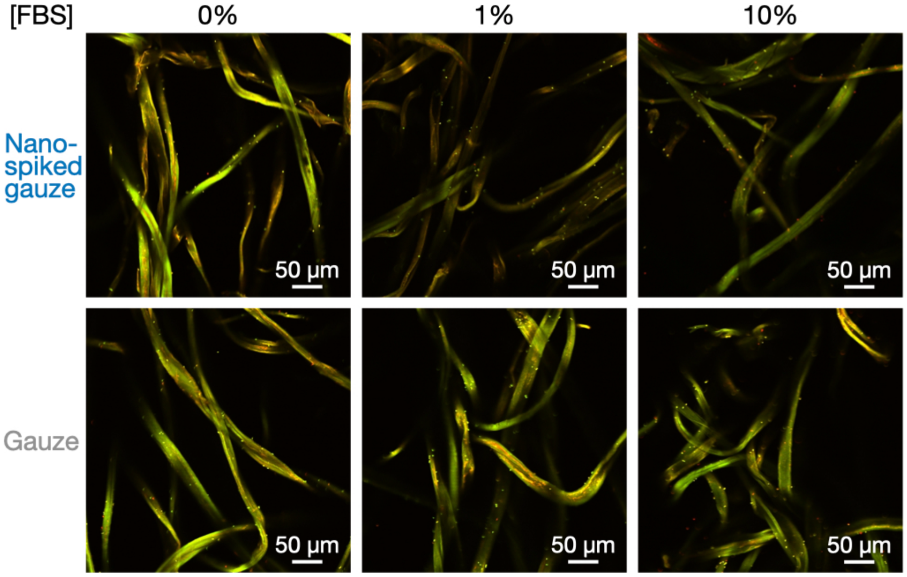


**Figure S7.** CLSM images of the nanospiked gauze and the raw gauze after incubation for 1 h with *E. coli* in the absence and presence of FBS.

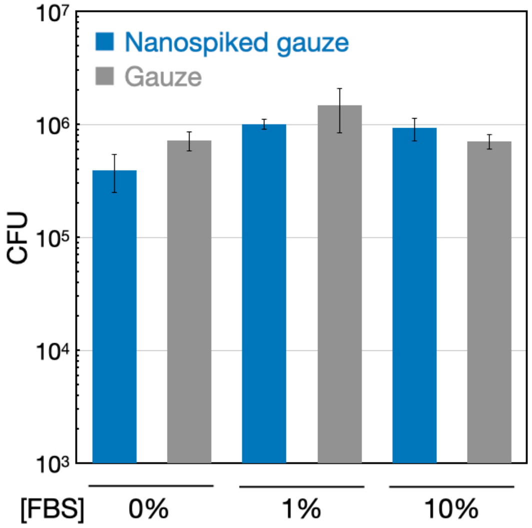


**Figure S8.** Colony counting assay for *E. coli* adhered to the nanospiked gauze and the raw gauze through incubation for 1 h in the absence and presence of FBS.

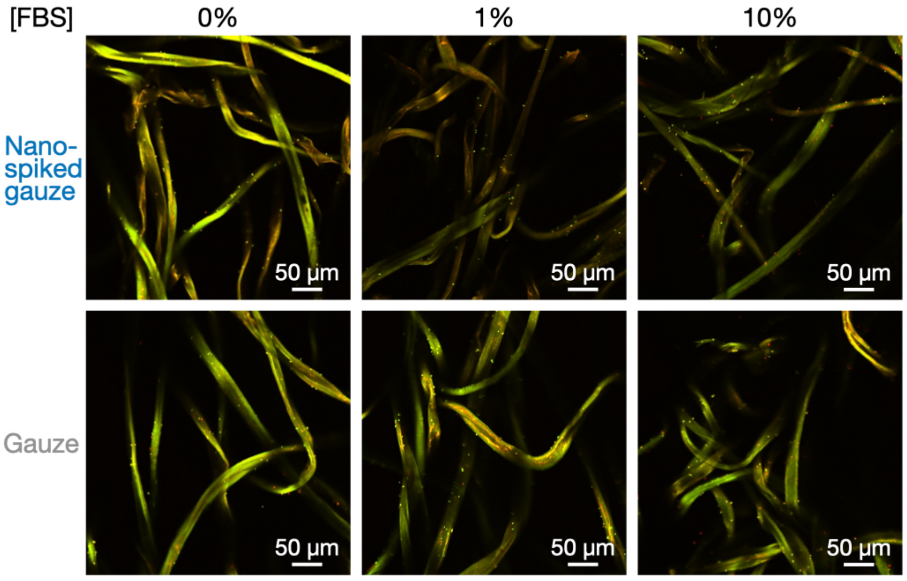


**Figure S9.** CLSM images of the nanospiked gauze and the raw gauze after incubation for 1 h with *P. aeruginosa* in the absence and presence of FBS.

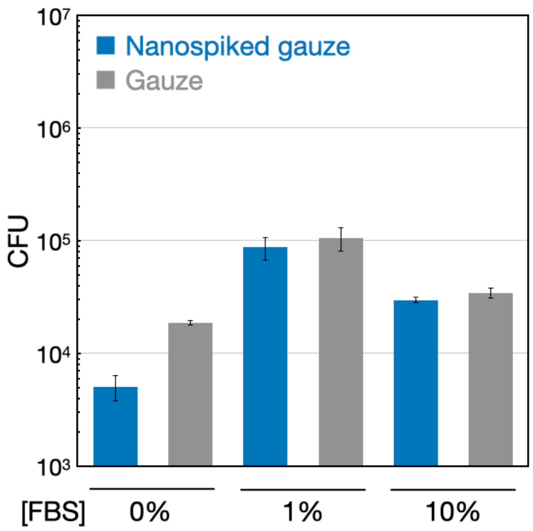


**Figure S10.** Colony counting assay for *P. aeruginosa* adhered to the nanospiked gauze and the raw gauze through incubation for 1 h in the absence and presence of FBS.

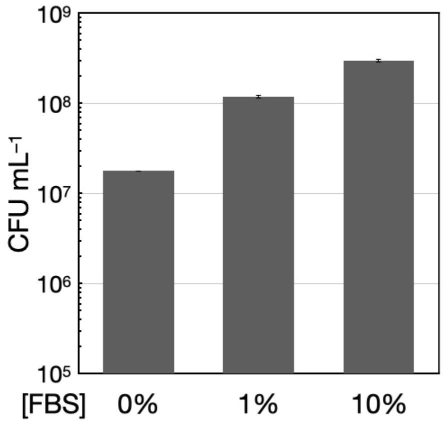


**Figure S11.** Concentrations of viable *P. aeruginosa* cells after incubation for 24 h in FBS solutions. The *P. aeruginosa* concentration before incubation was $1 \times 10^7$ CFU mL$^{-1}$.

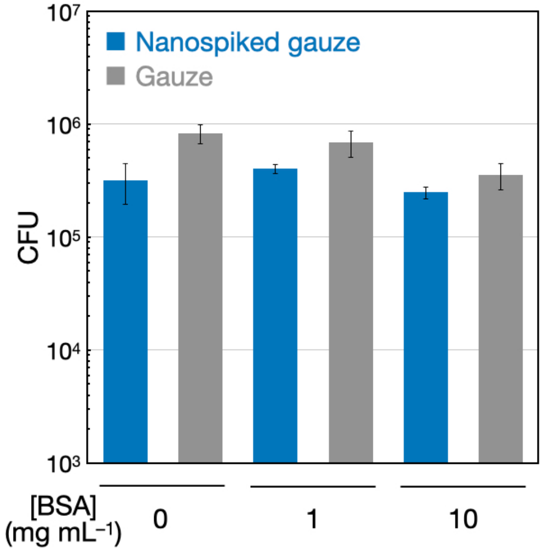


**Figure S12.** Colony counting assay for *E. coli* adhered to the nanospiked gauze and the raw gauze through incubation for 1 h in the absence and presence of BSA.

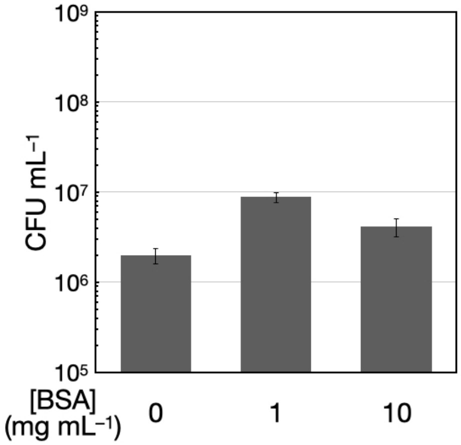


**Figure S13.** Concentrations of viable *E. coli* cells after incubation for 24 h in BSA solutions. The *E. coli* concentration before incubation was $1 \times 10^7$ CFU $mL^{-1}$.

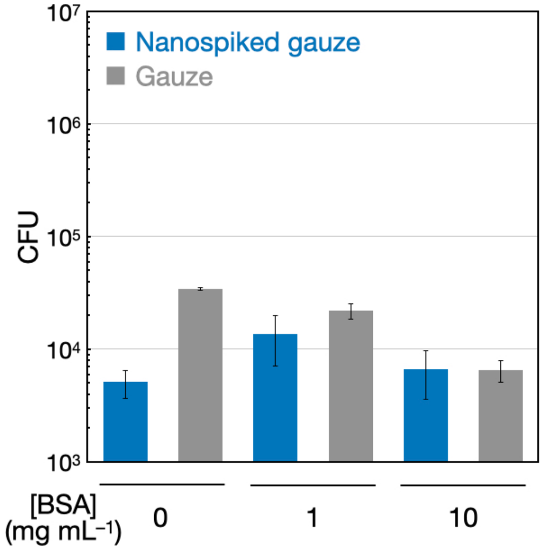


**Figure S14.** Colony counting assay for *P. aeruginosa* adhered to the nanospiked gauze and the raw gauze through incubation for 1 h in the absence and presence of BSA.

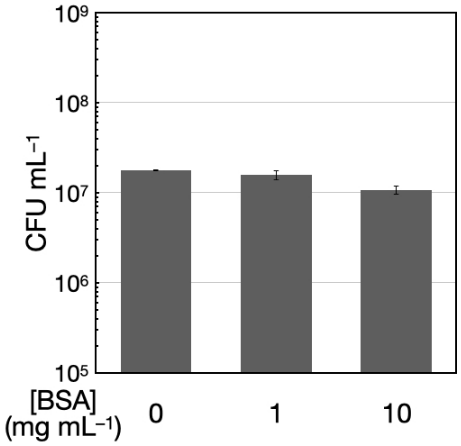


**Figure S15.** Concentrations of viable *P. aeruginosa* cells after incubation for 24 h in BSA solutions. The *P. aeruginosa* concentration before incubation was $1 \times 10^7$ CFU mL$^{-1}$.

REFERENCE

(1) Saito, A.; Miyazaki, H.; Fujie, T.; Ohtsubo, S.; Kinoshita, M.; Saitoh, D.; Takeoka, S. Therapeutic Efficacy of an Antibiotic-Loaded Nanosheet in a Murine Burn-Wound Infection Model. *Acta Biomater.* **2012**, *8* (8), 2932–2940. https://doi.org/10.1016/j.actbio.2012.04.019.
